# Is Pooled Data Analysis of Ventral and Incisional Hernia Repair Acceptable?

**DOI:** 10.3389/fsurg.2015.00015

**Published:** 2015-05-12

**Authors:** Ferdinand Köckerling, Christine Schug-Paß, Daniela Adolf, Wolfgang Reinpold, Bernd Stechemesser

**Affiliations:** ^1^Department of Surgery, Centre for Minimally Invasive Surgery, Vivantes Hospital Berlin, Academic Teaching Hospital of Charité Medical School, Berlin, Germany; ^2^StatConsult GmbH, Magdeburg, Germany; ^3^Department of Surgery, Wilhelmsburger Hospital Groß-Sand, Academic Teaching Hospital of University Hamburg, Hamburg, Germany; ^4^Hernia Center Cologne, Cologne, Germany

**Keywords:** incisional hernia, umbilical hernia, epigastric hernia, ventral hernia, primary ventral hernia, complications, recurrence

## Abstract

**Purpose:**

In meta-analyses and systematic reviews comparing laparoscopic with open repair of ventral hernias, data on umbilical, epigastric, and incisional hernias are pooled. Based on data from the Herniamed Hernia Registry, we aimed to investigate whether the differences in the therapy and treatment results justified such an approach.

**Methods:**

Between 1st September 2009 and 31st August 2013, 31,664 patients with a ventral hernia were enrolled in the Herniamed Hernia Registry. The implicated hernias included 16,206 umbilical hernias, 3,757 epigastric hernias, and 11,701 incisional hernias. Data on the surgical techniques, postoperative complication rates, and 1-year follow-up results were subjected to statistical analysis to identify any significant differences between the various hernia types.

**Results:**

The laparoscopic IPOM technique was used significantly more often for incisional hernia than for epigastric hernia, 31.3 vs. 24.0%, respectively, and was used for 12.9% of umbilical hernias (*p* < 0.0001). Likewise, the open technique with suturing of defect was used significantly more often for umbilical hernia than for epigastric hernia, 56.1 vs. 35.4%, respectively, and was used for 12.5% of incisional hernias (*p* < 0.0001). The postoperative complication rates of 3.2% for umbilical hernia and 3.5% for epigastric hernia were significantly lower than for incisional hernia, at 9.2% (*p* < 0.0001). That was also true for the reoperation rates due to postoperative complications, of 1.0 vs. 1.2 vs. 4.2% (*p* < 0.0001). The 1-year follow-up revealed significantly higher recurrence rates as well as rates of chronic pain needing treatment of 6.3 and 7.9%, respectively, for incisional hernia, compared with 4.1 and 4.3%, respectively, for epigastric hernia, and 2 and 1.9%, respectively, for umbilical hernia (*p* < 0.0001).

**Conclusion:**

Since significant differences were identified in the therapy and treatment results between umbilical hernia, epigastric hernia, and incisional hernia, scientific studies should be conducted comparing the various surgical techniques only for a single hernia type.

All the systematic reviews and meta-analyses published up to 2014 comparing laparoscopic with open repair of ventral hernias reported on studies in which data on primary ventral hernias (umbilical hernias, epigastric hernias) and incisional hernias were pooled and recurrences included. This meant that when analyzing the results no distinction was made between primary ventral hernias and incisional hernias (1–6) nor was any information given on the proportion of umbilical hernias, epigastric hernias, and incisional hernias identified in the entire patient group analyzed. It was only at the beginning of 2015 that Awaiz et al. (7) published the first meta-analysis and systematic review on laparoscopic vs. open incisional hernia repair.

Stirler et al. (8) were the first to point to significant differences in the results obtained for primary ventral hernias compared with incisional hernias. They concluded that continued pooling of data on primary ventral hernias and incisional hernias, as a combined entity, seemed incorrect. Based on data from the Herniamed Registry (9), this paper now aims to identify the differences between umbilical, epigastric, and incisional hernias in respect of the surgical techniques employed, postoperative outcome, and 1-year follow-up.

## Patients and Methods

Herniamed is a multicenter, Internet-based hernia registry in which 358 participating clinics and surgeons in private practice from Germany, Austria, and Switzerland (status: 31 August 2013) have prospectively registered their patients who had undergone hernia operations (9). This present analysis now examines the prospective data of all patients who had undergone open or laparoscopic umbilical, epigastric, or incisional hernia repair between 1st September 2009 and 31st August 2013. In total, 31,664 patients were enrolled (Table 1). The implicated hernias included 16,206 umbilical hernias (26% female), 3,757 epigastric hernias (48% female), and 11,701 incisional hernias (52% female). The age distribution shows a peak level for umbilical and epigastric hernias between 50 and 60 years and for incisional hernias between 70 and 80 years.

**Table 1:** **Comparison of the surgical techniques employed in umbilical, epigastric and incisional hernia repair**.

	Umbilical hernia *n* = 16,206	Epigastric hernia *n* = 3,757	Incisional hernia *n* = 11,701	*p*
Open suture	56.1% (*n* = 9,084) [55.3; 56.8]	35.4% (*n* = 1,330) [33.9; 36.9]	12.5% (*n* = 1,463) [11.9; 13.1]	<0.0001
Open sublay	–	18.5% (*n* = 695) [17.3; 19.7]	31.1% (*n* = 3,641) [30.3; 32.0]	<0.0001
Open onlay^a^	4.1% (*n* = 658) [3.8; 4.4]	3.3% (*n* = 123) [2.7; 3.9]	5.5% (*n* = 645) [5.1; 5.9]	<0.0001
Open IPOM	14.8% (*n* = 2,399) [14.3; 15.4]	10.9% (*n* = 410) [9.9; 11.9]	13.2% (*n* = 1,549) [12.6; 13.9]	<0.0001
Component separation^b^	1.5% (*n* = 250) [1.4; 1.7]	0.8% (*n* = 31) [0.6; 1.2]	1.6% (*n* = 184) [1.4; 1.8]	0.0022
Open others	10.7% (*n* = 1,726) [10.2; 11.1]	7.1% (*n* = 268) [6.3; 8.0]	4.8% (*n* = 562) [4.4; 5.2]	<0.0001
Laparoscopic IPOM	12.9% (*n* = 2,089) [12.4; 13.4]	24.0% (*n* = 900) [22.6; 25.3]	31.3% (*n* = 3,657) [30.4; 32.1]	<0.0001

*^a^*Post hoc*: overlapping of unadjusted 95% confidence intervals between umbilical and epigastric hernias*.

*^b^*Post hoc*: overlapping of unadjusted 95% confidence intervals between umbilical and incisional hernias*.

The following were calculated separately for each hernia type: the surgical techniques employed; the postoperative complication rates; reoperation rates due to postoperative complications; and the recurrence rates and rates of chronic pain needing treatment as identified on 1-year follow-up (9).

Using SAS 9.2 (SAS Institute Inc., Cary, NY, USA), a chi-square test was performed to investigate the differences between the various hernia types in respect of the surgical techniques employed, the postoperative complication rates, and the 1-year follow-up results. The results are expressed as a *p*-value (unadjusted analysis in each case up to the full significance level of 5%). Pairwise comparison of the individual hernia types was done on the basis of unadjusted estimate of an exact 95% confidence interval for each probability.

## Results

Analysis of the surgical techniques employed showed significant differences between the hernia types. For example, the laparoscopic IPOM technique was used significantly more often for incisional hernia, in 31.3% of cases (*p* < 0.0001), than for epigastric hernia, at 24.0%, and for umbilical hernia, at 12.9% (Table 1). Likewise, there was also a highly significant difference between umbilical hernia and epigastric hernia (*p* < 0.0001). The open technique with suturing of defect was also used significantly more often, in 56.1% of cases, for umbilical hernia than for epigastric hernia, at 35.4%, and for incisional hernia, at 12.5% (*p* < 0.0001). Likewise, there were significant differences in the use of the open Sublay mesh technique between epigastric hernia and incisional hernia, which was 18.5 vs. 31.1%, respectively (*p* < 0.0001), as well as in the open Onlay technique, which was 5.5% for incisional hernia, 4.1% for umbilical hernia, and 3.3% for epigastric hernia (*p* < 0.0001).

As regards the postoperative complication rates, comparable results were obtained for umbilical hernia, with a rate of 3.2%, and epigastric hernia, at 3.5%, but these differed significantly for incisional hernia with a rate of 9.2% (*p* < 0.0001) (Table 2). The same was true for the complication-related reoperation rates of 1.0% for umbilical hernia, 1.2% for epigastric hernia, and 4.2% for incisional hernia (*p* < 0.0001).

**Table 2:** **Comparison of the perioperative and 1-year outcome of umbilical, epigastric, and incisional hernia repair**.

	Umbilical hernia *n* = 16,206	Epigastric hernia *n* = 3,757	Incisional hernia *n* = 11,701	*p*
	1-year follow-up: *n* = 12,428/16,206	1-year follow-up: *n* = 2,895/3,757	1-year follow-up: 9,181/11,701	

1-year Follow-up rate	76.7% (*n* = 12,428/16,206)	77.1% (*n* = 2,895/3,757)	78.7% (*n* = 9,181/11,701)	
Postoperative complications^a^	3.2% (*n* = 515/16,206) [2.9; 3.5]	3.5% (*n* = 133/3,757) [3.0; 4.2]	9.2% (*n* = 1,075/11,701) [8.7; 9.7]	<0.0001
Reoperation rate for complications^a^	1.0% (*n* = 170/16,206) [0.9; 1.2]	1.2% (*n* = 44/3,757) [0.9; 1.6]	4.2% (*n* = 486/11,701) [3.8; 4.5]	<0.0001
Chronic pain needing treatment	1.9% (*n* = 240/12,428) [1.7; 2.2]	4.3% (*n* = 124/2,895) [3.6; 5.1]	7.9% (*n* = 729/9,181) [7.4; 8.5]	<0.0001
Recurrence rate	2.0% (*n* = 249/12,428) [1.8; 2.3]	4.1% (*n* = 119/2,895) [3.4; 4.9]	6.3% (*n* = 578/9,181) [5.8; 6.8]	<0.0001

*^a^*Post hoc*: overlapping of unadjusted 95% confidence intervals between umbilical and epigastric hernias*.

The differences in the treatment results between umbilical hernia, epigastric hernia, and incisional hernia were even more pronounced on 1-year follow-up. Accordingly, a significant difference was discerned in the recurrence rate of 2% for umbilical hernia, 4.1% for epigastric hernia, and 6.3% for incisional hernia (*p* < 0.0001). Equally, marked differences were noted in the rate of chronic pain needing treatment, which was 1.9% for umbilical hernia, 4.3% for epigastric hernia, and 7.9% for incisional hernia (*p* < 0.0001) (Table 2).

## Discussion

Based on the available registry data, it has been possible to identify significant differences in the utilization rates of the surgical techniques employed for the various ventral hernia types, i.e., umbilical hernia, epigastric hernia, and incisional hernia. Likewise, differences were found regarding the treatment results achieved for the various hernia types based on the postoperative complication rates, complication-related reoperation rates as well as recurrence rates, and rates of chronic pain needing treatment on 1-year follow-up. That highlights the fact that, when assessing the effectiveness of the various surgical techniques, the use of pooled analysis of the treatment results obtained for umbilical hernias, epigastric hernias, and incisional hernias can lead to incorrect results. Pooled data produce a result that is also dependent on the combination ratio between umbilical hernia, epigastric hernia, and incisional hernia. Therefore, the treatment results obtained for the various surgical techniques should only be compared for a single hernia type; that distinction was correctly made in the most recent meta-analysis, which focused only on incisional hernia, as conducted by Awaiz et al. (7). All the other meta-analyses and systematic reviews published to date have compared laparoscopic with open repair for ventral and incisional hernias (1–6). For these meta-analyses, it is not possible to ascertain what influence was exerted on the detailed results by the combination ratio of the hernia types experienced by the enrolled patient group. However, the study design to be used in future for prospective randomized trials and meta-analyses should ensure that comparison of the various surgical techniques employed is limited to one single clearly defined hernia type. As such, studies aimed at treatment optimization should be conducted separately for umbilical hernia, epigastric hernia, and incisional hernia.

Only in this way, a difference between the various surgical techniques can be properly identified for a specific ventral hernia type. In addition to prospective randomized comparative studies and meta-analyses, hernia registries can make an important contribution toward achieving that goal.

## Conflict of Interest Statement

Ferdinand Köckerling received grants for the Herniamed Registry from PFM Medical, Cologne; Johnson&Johnson, Norderstedt; BBraun, Tuttlingen; BARD, Karlsruhe; Dahlhausen, Cologne and MenkeMed, Munich. Christine Schug-Paß, Daniela Adolf, Wolfgang Reinpold and Bernd Stechemesser declare no conflict of interest.
